# Association between fatty acid intake and age-related macular degeneration: a meta-analysis

**DOI:** 10.3389/fnut.2024.1403987

**Published:** 2024-06-26

**Authors:** Yan Lee, Lok Lee, Li Zhang, Qing Zhou

**Affiliations:** ^1^Department of Ophthalmology, The First Affiliated Hospital of Jinan University, Guangzhou, China; ^2^International School of Jinan University, Guangzhou, China; ^3^School of Journalism and Communication, Jinan University, Guangzhou, China

**Keywords:** dietary fatty acids (FAs), long-chain omega-3 polyunsaturated fatty acids (omega-3 LCPUFAs), docosahexaenoic acid (DHA), eicosapentaenoic acid (EPA), trans-fatty acid (TFA), age-related macular degeneration (AMD), meta-analysis

## Abstract

**Objective:**

The association of age-related macular degeneration (AMD) with the intake of high and low fatty acids (FAs), respectively, remains controversial. To this end, we performed a comprehensive meta-analysis of all the existing studies on the association of various intake levels of FA subtypes with AMD to determine these associations.

**Methods:**

A systematic search of PubMed, Web of Science, Cochrane Library, and EMBASE databases was conducted from inception to September 2023. To compare the highest and lowest groups, odds ratio (OR) with 95% confidence intervals (CIs) was analyzed with a random-effects model/fixed-effects model.

**Results:**

A high intake of omega-3 LCPUFAs (OR:0.67; 95%CI:[0.51, 0.88]; *p* = 0.004), DHA (OR:0.80; 95%CI:[0.70, 0.90]; *p* < 0.001), EPA (OR:0.91; 95%CI:[0.86, 0.97]; p = 0.004), and simultaneous intake of DHA and EPA (OR:0.79; 95%CI:[0.67, 0.93]; *p* = 0.035) significantly reduced the risk of overall AMD. Conversely, a high intake of trans-FAs (OR: 2.05; 95%CI: [1.29, 3.25]; *p* = 0.002) was significantly related to an increased risk of advanced AMD compared to the low-intake group. The subgroup analysis results are shown in the articles.

**Conclusion:**

Increasing dietary intake of omega-3 LCPUFAs, specifically DHA, and EPA, or the simultaneous intake of DHA and EPA, is significantly associated with a reduced risk of overall AMD. Various subtypes of omega-3 also have a significant association with a reduced risk of different stages of AMD. The high intake of trans-fatty acids (TFAs) is significantly and positively correlated with the risk of advanced AMD. This could further support the idea that consuming foods rich in omega-3 LCPUFAs and reducing consumption of foods rich in TFAs may prevent AMD.

**Systematic review registration:**

https://www.crd.york.ac.uk/prospero/, identifier CRD42023467227.

## 1 Introduction

Age-related macular degeneration (AMD) is the most prevalent cause of irreversible vision loss in older patients and ranks as the fourth leading cause of blindness, mainly affecting people over 55 years of age (1, 2). With the acceleration of population aging, the number of AMD patients has been increasing. According to estimates, the number of individuals with AMD in the world accounts for 8.69% of the world population and is expected to reach 288 million by 2040, among which more than 60% of the AMD patients are from Asia, imposing huge financial and policy burdens worldwide (1, 3, 4). Although several effective therapeutic drugs are available, repeated and frequent injections and doctor visits increase the financial burden on the healthcare system and patients. Furthermore, treatment-related adverse effects, such as endophthalmitis, retinal detachment, and traumatic lens injury, can reduce patient compliance and further compromise vision as the disease progresses (4–6). Therefore, preventing the development of the disease and delaying its progression is recommended for a better prognosis (4).

In addition to some recognized risk factors for AMD such as age, gender, race, and smoking (1, 3), cumulative oxidative damage to retinal pigment epithelial (RPE) cells is also reported as a major contributor to AMD (4, 7). Therefore, the relationship between lipids and AMD has attracted increasing attention. As people grow older, lipofuscin continues to accumulate in RPE cells and cannot be degraded, which leads to cellular hypoxia and chronic inflammation, thereby resulting in cumulative oxidative damage to cells (4).

Dietary fatty acids (FAs) include saturated fatty acids (SFAs), polyunsaturated fatty acids (PUFAs), and monounsaturated fatty acids (MUFAs). They are vital sources of energy involved in lipogenesis, glycolysis, and protein synthesis (8). PUFAs are primarily obtained from food and are beneficial to anti-inflammatory and antithrombotic processes, as well as for maintaining vision, cognitive function, and glucose and lipid metabolism (8). Long-chain omega-3 polyunsaturated fatty acids (omega-3 LCPUFAs), as the main structural components of the retina, have anti-angiogenic, anti-proliferation of the blood vessel, and neuroprotective effects in terms of the pathogenic factors and processes of proliferative and degenerative retinal diseases. They also protect against oxygen toxicity, inflammation, and age-related retinal damage (9, 10). Many studies have shown that omega-3 FAs are believed to lower the risk of AMD, with a clear difference between high and low intake levels of docosahexaenoic acid (DHA, C22: 6 n-3) in protecting against the development of AMD (11–16), while a small number of studies indicate no difference (17–21). Simultaneously, omega-6 fatty acids are also considered to have a protective effect on the progression of AMD in some studies, but the results of different studies are conflicting; one study suggested that the high and low intake levels of omega-6 were significantly different in reducing the incidence of AMD (22). Another study found a significant difference in increasing the incidence of AMD between the high-omega-6- and low-omega-6-intake groups (23), and three studies concluded that the high and low intake levels of omega-6 did not affect the incidence of AMD (13, 18, 24). Other FA subtypes face a similar situation.

There are many types of FAs, and the effects of various intake levels of different FA subtypes seem to be different in the development and progression of AMD, and the study results are always different among studies, making clinicians confused about how much level of intake of FAs can prevent and delay the development of AMD. To this end, a comprehensive meta-analysis of all existing studies on the association between different intake levels of various FA subtypes and AMD was performed in our study, to investigate the association of the intake of various FA subtypes with the development and progression of AMD.

## 2 Methodology

The Meta-analysis of Observational Studies in Epidemiology (MOOSE) protocol was followed in the design, performing, and analysis of our research, and the Preferred Reporting Items for Systematic Reviews and Meta-Analyses (PRISMA) statement was followed in the reporting of the results (25, 26). The protocol of our systematic review has been registered with the International Prospective Register of Systematic Reviews (PROSPERO; ID: CRD42023467227). The current meta-analysis was carried out to analyze the risk factors of AMD, and the exposure factors examined were various FA subtypes. Cohort studies, case–control studies, and cross-sectional studies investigating AMD were included in the present study.

### 2.1 Literature search

For studies on AMD and dietary FAs, a systematic search of PubMed, Web of Science, Cochrane Library, and EMBASE databases was conducted from the inception of each database up to May 2024. The keywords used for the literature search included fatty acid, age-related macular degeneration, and fish Mediterranean diet, and the search strategy is detailed in the Supplementary material. There were no restrictions on study type, language, or country.

### 2.2 Selection criteria

We included the following eligible studies in our meta-analysis: (1) studies in which the early, intermediate, and advanced AMD were adequately defined as follows: Normal aging was defined as only having drupelets (small drusen ≤63 μm), while early AMD was defined as having drusen measuring between 63 μm and 125 μm, with no pigment abnormalities associated with AMD. The intermediate AMD was defined as having drusen >125 μm and pigment abnormalities associated with AMD, or without AMD-related pigment abnormalities. Occurrence of neovascular AMD (wet/exudative AMD) or geographic atrophy (dry AMD) indicated advanced AMD (27). The drusen refers to a cell-free, lipid-rich deposit under the RPE (4). (2) All observational studies, such as case–control studies, prospective/retrospective cohort studies, and cross-sectional studies; (3) studies where the intake of different dietary FA subtypes was examined as exposures; (4) studies with data on the association of the highest and lowest levels of FA intake with the risk of AMD in different stages. We excluded the following studies: non-human studies, non-English publications, case reports, overlapping reports, reviews, studies lacking sufficient data, meta-analyses, and publications of which the full texts could not be found.

### 2.3 Data extraction

Two independent researchers (L. L and Y. L) searched the literature and extracted the data required, separately. First, the two researchers conducted a preliminary screening of the title and abstract separately and then evaluated all eligible studies by reading the full texts, and disagreement, if any, was resolved through group discussion. After the studies to be included were identified, the following data were extracted separately from each of the included publications: first author, year of publication, study location, study type, gender as well as age of participants, sample size (cases and number of participants), method of assessing intake of dietary FAs, type of dietary FAs, criteria for diagnosing AMD, type of AMD studied, adjusted covariates multivariate analysis, and 95%Cl risk assessment. If multiple multivariate adjustment models were used to report risk assessment in the original studies, data from the model with the most adjustments were extracted.

### 2.4 Quality assessment

The widely used Newcastle–Ottawa Scale (NOS) was employed to evaluate the quality of case–control studies and cohort studies, and the criteria recommended by the Agency for Healthcare Research and Quality (AHRQ) were adopted to evaluate the cross-sectional studies. The included studies were comprehensively evaluated using the NOS in terms of outcome (cohort studies) or exposure (case–control studies), study selection, and comparability. Each item could be given a maximum of 1 point, and for comparison, some items could be given a maximum of 2 points. The quality of the studies was evaluated as per the following standards: 0 ~ 3 for low quality, 4 ~ 6 for medium quality, and ≥ 7 for high quality (28). The 11-item checklist recommended by the AHRQ includes the definition of information source, period to identify patient and continuity of patient identification, blinding of personnel, inclusion and exclusion criteria, quality assurance assessment, confusing and missing data, and patient response rate and completeness. The maximum score of each item is 1 point. The quality of the studies was evaluated as per the following standards: 0 ~ 3 for low quality, 4 ~ 7 for medium quality, and ≥ 8 for high quality (28).

### 2.5 Statistical analysis

The OR and 95% CI were believed to be common indicators in the current meta-analysis for the association of each type of dietary FA with the risk of AMD in the studies. Pooled effect estimates were reported with 95% CIs. Before the pooled effect was assessed, the Q test and I^2^ test were conducted to examine the heterogeneity among the studies. I^2^ < 50% and *p* > 0.1 indicated the presence of small heterogeneity, and a fixed-effects model was used for meta-analysis; otherwise, a random-effects model was used. In addition, a sensitivity analysis was carried out for each risk factor by eliminating each study from the overall analysis. Egger’s test was conducted to estimate the publication bias for FA groups involving 10 or more studies, and *p* > 0.05 indicated that the publication bias was not significant. The publication bias, if any, was corrected to evaluate whether there were consistent results after data with biases were removed.

## 3 Results

### 3.1 Study selection process

**Figure 1:**
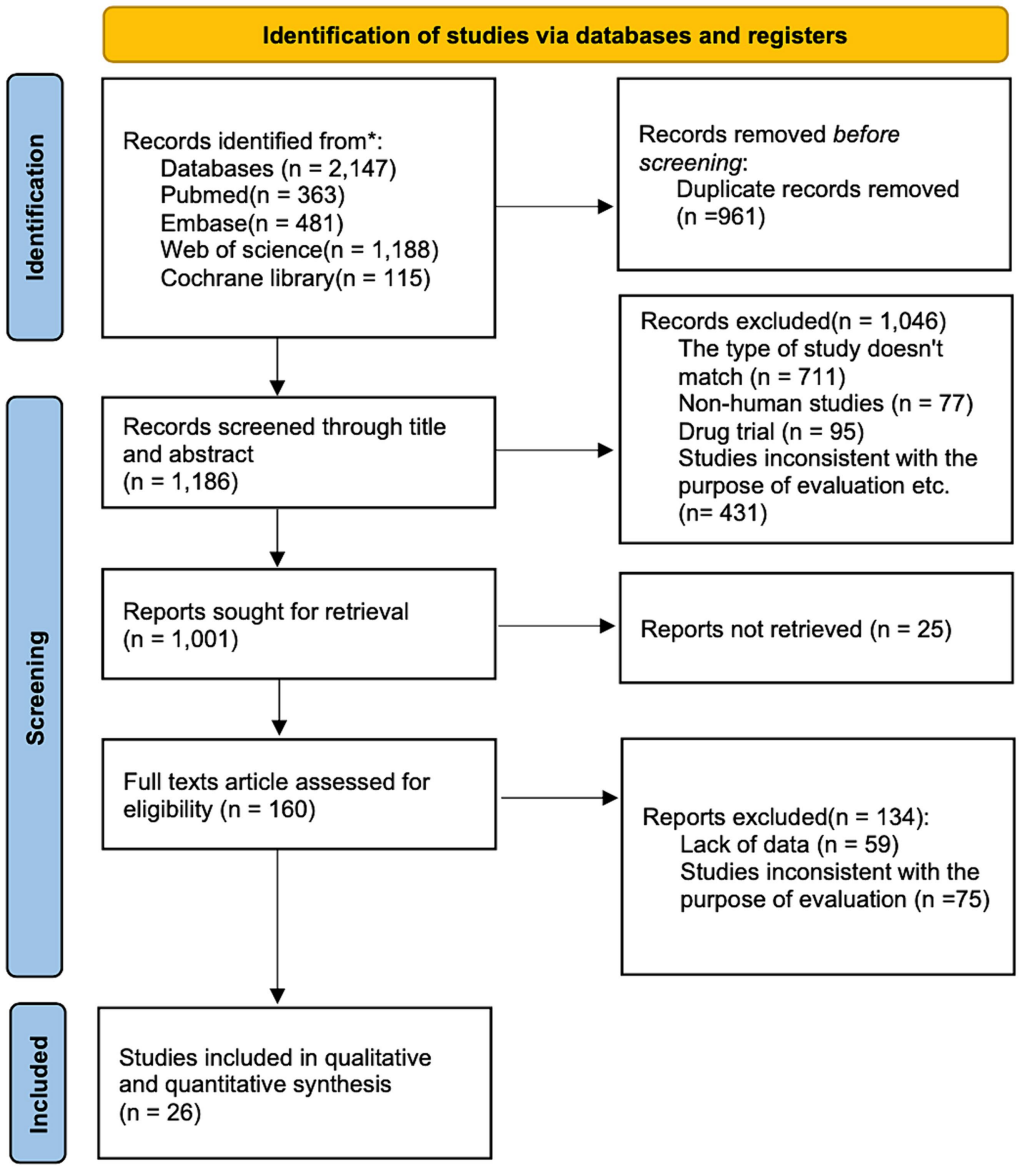
Flowchart of literature selection and selection strategy.

Our initial screening identified 2,147 records (115 from Cochrane Library, 481 from Embase, 363 from PubMed, and 1,188 from Web of Science) as shown in Figure 1. We excluded 2,121 duplicates and other records not meeting the above inclusion criteria, and finally, 26 studies (14 cohort studies, 3 case–control studies, and 9 cross-sectional studies) were included, involving a total of 241,151 participants.

### 3.2 Characteristics of the included studies

Table 1 summarizes the descriptive characteristics of the included literature. In the 26 studies included in our research, data on dietary fat intake were based on the Brief Self-administered Diet History Questionnaire (BDHQ) in one study and the Food Frequency Questionnaire (FFQ) in the remaining 25 studies. Of the 26 studies, 15 were carried out in the United States, 5 in Australia, 2 in Europe, 1 in Hong Kong (China), and 3 in Japan.

**Table 1:** Characteristics of the included studies (*n* = 26).

Characteristics of participants				Characteristics of exposure		Case of AMD	Study quality evaluation^a^
Author, publication year	Study design	Country	Age	Dietary assessment methods	Exposure		
Mares-Perlman et al., 1995	Retrospective cohort study	The United States	45–84	FFQ	Total fat; Saturated fat Oleate Linoleate Cholesterol	314 early AMD, 30 late AMD	High
Smith et al., 2000	Cross-sectional study	Australia	≥49y	FFQ	Total fat Saturated fat Cholesterol Polyunsaturated fat Monounsaturated fat	182 early AMD, 46 late AMD	High
Cho et al., 2001	Perspective cohort study	The United States	≥ 50y	FFQ	Total fat; Saturated fat Monounsaturated fat Polyunsaturated fat (linoleic acid; linolenic acid; arachidonic acid; eicosapentaenoic acid; docosahexaenoic acid) Trans Unsaturated fat	567 AMD (any stages)	High
Seddon et al., 2003	Perspective cohort study	The United States	≥ 60y	FFQ	Saturated fat Monounsaturated fat Polyunsaturated fat Trans unsaturated fat	101 late AMD	High
Chua et al., 2006	Perspective cohort study	Australia	≥ 49y	FFQ	Total dietary fat Saturated fa Monounsaturated fat Polyunsaturated fat (total n-3 polyunsaturated fatty acids; a-linolenic acid; long-chain n-3 polyunsaturated fatty acids; total n-6 polyunsaturated fatty acids; linoleic acid; arachidonic acid) Trans-unsaturated fat	158 early AMD, 26 late AMD	High
Robman et al., 2007	Cross-sectional study	Australia	74 ± 7	FFQ	Omega-3 FA	83 AMD (any stages)	High
SanGiovanni et al., 2007	Case–control study	The United States	60–80	AREDS FFQ	Omega-3 FA (a-linolenic acid; 18:3–3) eicosapentaenoic acid (20:5 w-3) docosahexaenoic acid (22:6 w-3)) Total w-3 LCPUFAs w-6 Fatty acids (linoleic acid (18:2w-6; arachidonic acid (20:4 w-6)) Monounsaturated fatty acids Saturated fatty acids Dietary cholesterol	657 AMD (any stages)	High
Delcourt et al., 2007	Perspective cohort study	France	>60	FFQ	Total fat Saturated fatty acids Monounsaturated fatty acids Polyunsaturated FA	46 early AMD, 12 late AMD	High
Augood et al., 2008	Cross-sectional study	Europe	≥ 65	FFQ	DHA EPA	105 NV-AMD	High
Chiu et al., 2009	Cross-sectional study	The United States	55–80	FFQ	DHA EPA	4,454 early AMD, 747 late AMD	High
Chong et al., 2009	Perspective cohort study	Australia	40–69	FFQ	Total fat Polyunsaturated fat Monounsaturated fat Saturated fat Trans fat Oleic acid Linoleic acid Arachidonic acid Omega-3 fatty acids Marine omega-3 fatty acids Eicosapentaenoic acid Docosahexaenoic acid Alpha-linolenic acid	1921 early AMD, 77 late AMD	High
Parekh et al., 2009	Perspective cohort study	The United States	50–79	FFQ	Total fat Saturated fat Monounsaturated fat Omega-6 PUFA Omega-3 PUFA;	1787 intermediate AMD	High
SanGiovanni et al., 2009	Perspective cohort study	The United States	55–80	FFQ	DHA EPA	364 central geographic atrophy, 583 NV-AMD	High
Tan et al., 2009	Perspective cohort study	Australia	>49	FFQ	Saturated FA Monosaturated FA PUFAs Total fat Tans-unsaturated FA Total omega-3 PUFAs Long-chain omega-3 PUFAs Alpha-linolenic acid Total omega-6 PUFAs Linoleic acid Arachidonic acid	220 early AMD, 59 late AMD	High
Christen et al., 2011	Perspective cohort study	The United States	>45	FFQ	Omega-3 FA Omega-6 fatty acid ALA; AA; LA; DPA; DHA; EPA	235 AMD (any stages)	High
Ho et al., 2011	Perspective cohort study	The Netherlands	≥ 55	FFQ	EPA + DHA	517 early AMD	High
Aoki et al., 2016	Case–control study	Japan	AMD: 73.5 ± 7.1 Control: 73.1 ± 5.6	BDHQ	Omega-3 FA	157 AMD (any stages)	High
Wu et al., 2017	Perspective cohort study	The United States	≥ 50	FFQ	ALA Omega-3 FA	1,589 intermediate AMD, 1,356 late AMD	High
Wu et al., 2017	Perspective cohort study	The United States	≥ 50	FFQ	EPA DHA;		High
Ng et al., 2019	Case–control study	Hong Kong	Exudative AMD patient: 73.7 ± 10.2 Control: 67.1 ± 9.3	FFQ	Omega-3 FA Omega-6 FA	99 exudative AMD	High
Roh et al., 2020	Cross-sectional study	The United States	AMD:74.2 ± 7.9 Control: 68.3 ± 6.7	FFQ	Trans fat Saturated fat PUFA Omega-3 FA Omega-6 FA MUFA	90 early AMD, 201 intermediate AMD 95 late AMD	High
Sasaki et al., 2020	Cross-sectional study	Japan	Early AMD: 62.0 control: 65.3	FFQ	Total fat Saturated fatty acid Monounsaturated FA Polyunsaturated FA n3-polyunsaturated FA; n6-polyunsaturated FA n3-highly unsaturated FA SFA (total, HDL, LDL cholesterol, triglycerides)	447 early AMD	High
Edo et al., 2021	Cross-sectional study	The United States	Early AMD: 66.5 ± 10.3 control: 60.4 ± 13.7	FFQ	SFA; PUFA; cholesterol	111 early AMD,	High
Elmore et al., 2022	Cross-sectional study	The United States	≥ 70	FFQ	EPA, DHA, ALA, LA, AA	378 AMD (any stages)	High
Karger et al., 2022	Perspective cohort study	The United States	45–84	FFQ	DHA, EPA, DHA + EPA	214 early AMD	High
Yasukawa et al., 2023	Cross-sectional study	Japan	62.4 ± 9.4	FFQ	Total fat Saturated fatty acid Monounsaturated fatty acids Polyunsaturated fatty acids Omega-3 FA Linolenic acid Eicosapentaenoic acid (EPA) Docosahexaenoic acid (DHA) Omega-6 FA	1,421 early AMD, 906intermediate AMD 29 late AMD	High

a Study quality for cohort and case–control studies was assessed with the use of Newcastle–Ottawa Scale, and cross-sectional studies were assessed with the use of the Agency for Healthcare Research and Quality (AHRQ).

### 3.3 Quality assessment results of the included studies

We adopted the NOS and AHRQ to evaluate the quality of different types of studies. The cohort and case–control studies were all awarded ≥7 points, and all the cross-sectional studies were awarded>8 points, suggesting that the included literature was of high quality (Tables 2–4). The FFQ and BDHQ used in these studies to assess dietary FA levels applied to large cohorts and provided information on a variety of foods. However, these tools have several limitations, including incorrect reporting of diet, which can lead to misclassification of dietary intake and/or amount. Therefore, studies evaluated using the NOS were all given a score of 0 for “ascertainment of exposure.”

**Table 2:** Newcastle–Ottawa scale (cohort) for 14 studies included in this meta-analysis.

Study (Author+Year)	Cohort Selection				Comparability	Result			Quality Score
	Representativeness of the Exposed Cohort (high representativeness/good representativeness)	Selection of the Non-Exposed Cohort (from the same population and community as the exposed cohort)	Ascertainment of Exposure (Strict and accurate records (such as surgical records)/structured questionnaires)	Demonstration That Outcome of Interest Was Not Present at Start of Study (Yes)	Comparability of Cohorts on the Basis of the Design or Analysis (Select and analyze controls based on the most important factor/Select and analyze controls based on other important factors such as the second most important factor)	Assessment of Outcome (Independent, blinded measurement or assessment/based on reliable records)	Was Follow-Up Long Enough for Outcomes to Occur (Yes)	Adequacy of Follow-Up of Cohorts (Adequate follow-up, all study subjects are followed up/follow-up rate > 90%)	
Mares-Perlman et al., 1995	1	1	0	1	2	1	1	1	8
Cho et al., 2001	1	1	0	1	2	1	1	1	8
Seddon et al., 2003	1	1	0	1	2	1	1	1	8
Chua et al., 2006	1	1	0	1	2	1	0	1	7
Delcourt et al., 2007	1	1	0	1	2	1	0	1	7
Chong et al., 2009	1	1	0	1	2	1	1	1	8
Parekh et al., 2009	1	1	0	1	2	1	1	1	8
SanGiovanni et al., 2009	1	1	0	1	2	1	1	1	8
Tan et al., 2009	1	1	0	1	2	1	1	1	8
Christen et al., 2011	1	1	0	1	2	1	1	1	8
Ho et al., 2011	1	1	0	1	2	1	1	1	8
Wu et al., 2017	1	1	0	1	2	1	1	1	8
Wu et al., 2017	1	1	0	1	2	1	1	1	8
Karger et al., 2022	1	1	0	1	2	1	1	1	8

**Table 3:** Newcastle-Ottawa scale (case–control) for three studies included in this meta-analysis.

Study(Author+Year)	Selection of Cases and Controls				Comparability	Exposure			Quality Score
	Is the Case Definition Adequate? (The definition and diagnosis of the disease are correct, independent and valid)	Representativeness of the Cases (Contiguous cases, or cases of representativeness)	Selection of Controls (community control)	Definition of Controls (No history of disease of interest)	Comparability of Cases and Controls (Select and analyze controls based on the most important factors)	Ascertainment of Exposure (Reliable records; e.g., surgical records)	Are the methods the same for cases and controls? (Yes)	Non-Response Rate (Non-response rates were the same between the two groups)	
SanGiovanni et al., 2007	1	1	0	1	2	0	1	1	7
Aoki et al., 2016	1	1	1	1	2	0	1	1	8
Ng et al., 2019	1	1	0	1	2	0	1	1	7

**Table 4:** Agency for healthcare research and quality (AHRQ) checklist (cross-sectional) for nine studies included in this meta-analysis.

Study (Author+Year)	Yasukawa et al., 2023	Elmore et al., 2022	Edo et al., 2021	Sasaki et al., 2020	Roh et al., 2020	Chiu et al., 2009	Augood et al., 2008	Robman et al., 2007	Smith et al., 2000
Define the source of information (Survey, record review)	1	1	1	1	1	1	1	1	1
List inclusion and exclusion criteria for exposed and unexposed subjects (cases and controls) or refer to previous publications	1	1	0	1	1	1	1	1	1
Indicate time period used for identifying patients	1	1	1	1	1	1	1	1	0
. Indicate whether or not subjects were consecutive if not population-based	1	1	1	1	1	1	1	1	1
Indicate if evaluators of subjective components of study were masked to other aspects of the status of the participants	1	1	1	1	1	1	1	1	1
Describe any assessments undertaken for quality assurance purposes (e.g., test/retest of primary outcome measurements)	1	1	1	1	1	1	1	1	1
Explain any patient exclusions from analysis	1	1	1	1	1	1	1	0	0
Describe how confounding was assessed and/or controlled	1	1	1	1	1	1	1	1	1
If applicable, explain how missing data were handled in the analysis	1	1	1	1	1	1	1	1	1
Summarize patient response rates and completeness of data collection	1	1	1	1	1	1	1	1	1
Clarify what follow-up, if any, was expected and the percentage of patients for which incomplete data or follow-up was obtained	1	1	1	1	1	1	1	1	1
Quality Scores	11	11	10	11	11	11	11	10	9

### 3.4 Meta-analysis

#### 3.4.1 Total fat

In total, 10 of the included studies analyzed the relationship between total fat intake and the risk of overall AMD, and the statistical heterogeneity among the studies was large (I^2^ = 59.1%, P_H_ = 0.001) (18, 20, 21, 23, 24, 29–33). We compared the high and low intake levels of total fat and found that the increased intake of total fat did not influence the risk of overall AMD, early AMD, intermediate AMD, or advanced AMD.

#### 3.4.2 Trans-fatty acids

**Figure 2:**
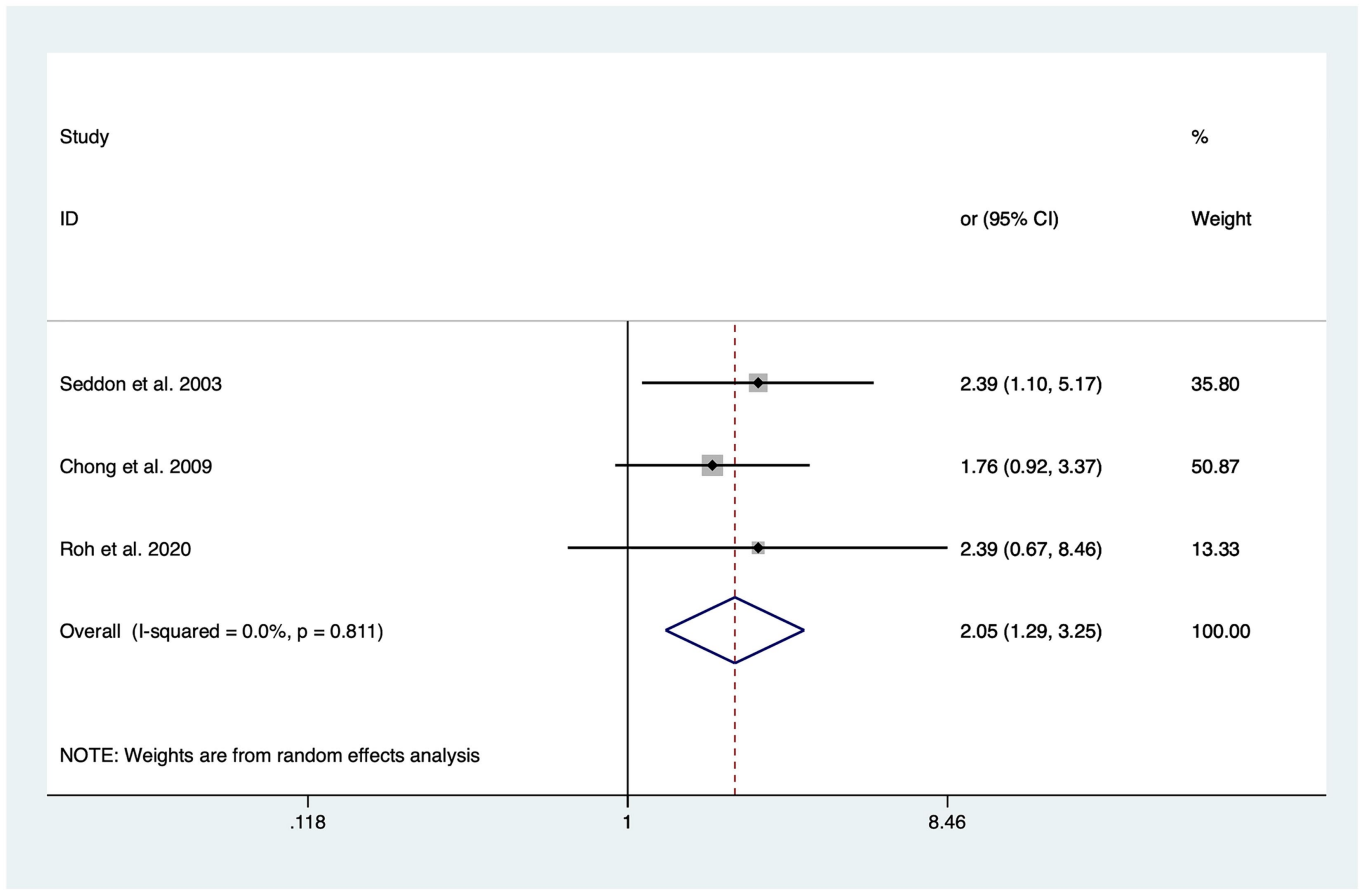
Forest plot of the odd risk (OR) of advanced AMD for the highest vs. lowest level intake of trans-fatty acid in studies.

In total, six of the included studies analyzed the association of intake of trans-fatty acids (TFAs) with the risk of overall AMD, and the statistical heterogeneity among the studies was large (I^2^ = 63.6%, P_H_ = 0.005) (20–22, 31, 32, 34). The risk of advanced AMD (OR: 2.05; 95%CI: [1.29, 3.25]; *p* = 0.002) was significantly increased through a high intake of TFAs as shown in Figure 2. Although increasing the intake of TFAs did not affect the risk of overall AMD, early AMD, and intermediate AMD, the OR value increased with the progression of AMD in different stages.

#### 3.4.3 SFAs

In total, 14 of the included studies analyzed the relationship between intake of SFAs and the risk of overall AMD, and the statistical heterogeneity among the studies was large (I^2^ = 55.5%, P_H_ = 0.001) (15, 18, 20, 24, 29–35). We compared the high and low intake levels of SFAs and found that increasing the intake of SFAs did not influence the risk of overall AMD, early AMD, intermediate AMD, or advanced AMD.

#### 3.4.4 MUFAs

In total, 11 of the included studies analyzed the relationship between intake of MUFAs and the risk of overall AMD, and the statistical heterogeneity among the studies was large (I^2^ = 61.3%, P_H_ < 0.001) (15, 18, 20–24, 30–34). We compared the high and low intake levels of MUFAs and found that increasing the intake of MUFAs did not influence the risk of overall AMD, early AMD, intermediate AMD, or advanced AMD.

#### 3.4.5 PUFAs

In total, 11 of the included studies analyzed the relationship between intake of PUFAs and the risk of overall AMD, and the statistical heterogeneity among the studies was large (I^2^ = 53.4%, P_H_ = 0.006) (18, 20–22, 24, 30–35). We compared the high and low intake levels of PUFAs and found that increasing the intake of PUFAs did not influence the risk of overall AMD, early AMD, or advanced AMD.

#### 3.4.6 Omega-3 PUFAs family

**Figure 3:**
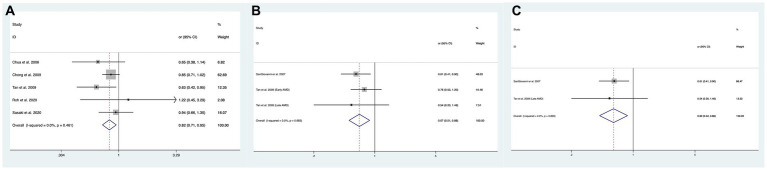
Forest plot of the odds risk (OR) of AMD for the highest vs. lowest level intake of omega-3 family in studies. **(A)** The odds risk of omega-3 PUFAs and early AMD; **(B)** the odds risk of omega-3 LCPUFAs and overall AMD; **(C)** the odds risk of omega-3 LCPUFAs and advanced AMD.

In total, 10 of the included studies analyzed the association of the intake of omega-3 PUFAs with the risk of overall AMD, and the statistical heterogeneity among the studies was large (I^2^ = 68.3%, P_H_ = < 0001) (13, 18, 21–24, 31, 32, 36, 37). We compared the high and low intake levels of omega-3 PUFAs and observed a significant difference between the intake of high and low omega-3 PUFAs in the risk of early AMD (OR: 0.82; 95%CI: [0.71, 1.05]; *p* = 0.148) as shown in Figure 3A. In contrast, increasing the intake of omega-3 PUFAs showed no difference in the risk of overall AMD, intermediate AMD, or advanced AMD compared to the low-intake group.

#### 3.4.7 Omega-3 LCPUFAs and alpha-linolenic acid

In total, two of the included studies analyzed the association of intake of omega-3 LCPUFAs with the risk of overall AMD, and no statistical heterogeneity was observed among studies (I^2^ = 0.0%, P_H_ = 0.683) (15, 32). We compared the high and low intake levels of omega-3 LCPUFAs and observed a significant difference in the risk of overall AMD (OR:0.67; 95%CI: [0.51, 0.88]; *p* = 0.004) and advanced AMD (OR:0.60; 95%CI: [0.42, 0.87]; *p* = 0.006) between the increased intake of omega-3 LCPUFAs and the low-intake group as shown in Figures 3B,C.

In addition, eight studies analyzed the relationship between ALA intake and the risk of overall AMD, and there was large statistical heterogeneity among studies (I^2^ = 55.0%, P_H_ = 0.01) (13, 15–17, 21, 31, 32, 38). The high intake of ALA had no association with overall AMD, early AMD, intermediate AMD, or advanced AMD.

#### 3.4.8 DHA and eicosapentaenoic acid (EPA, C20: 5 n-3)

**Figure 4:**
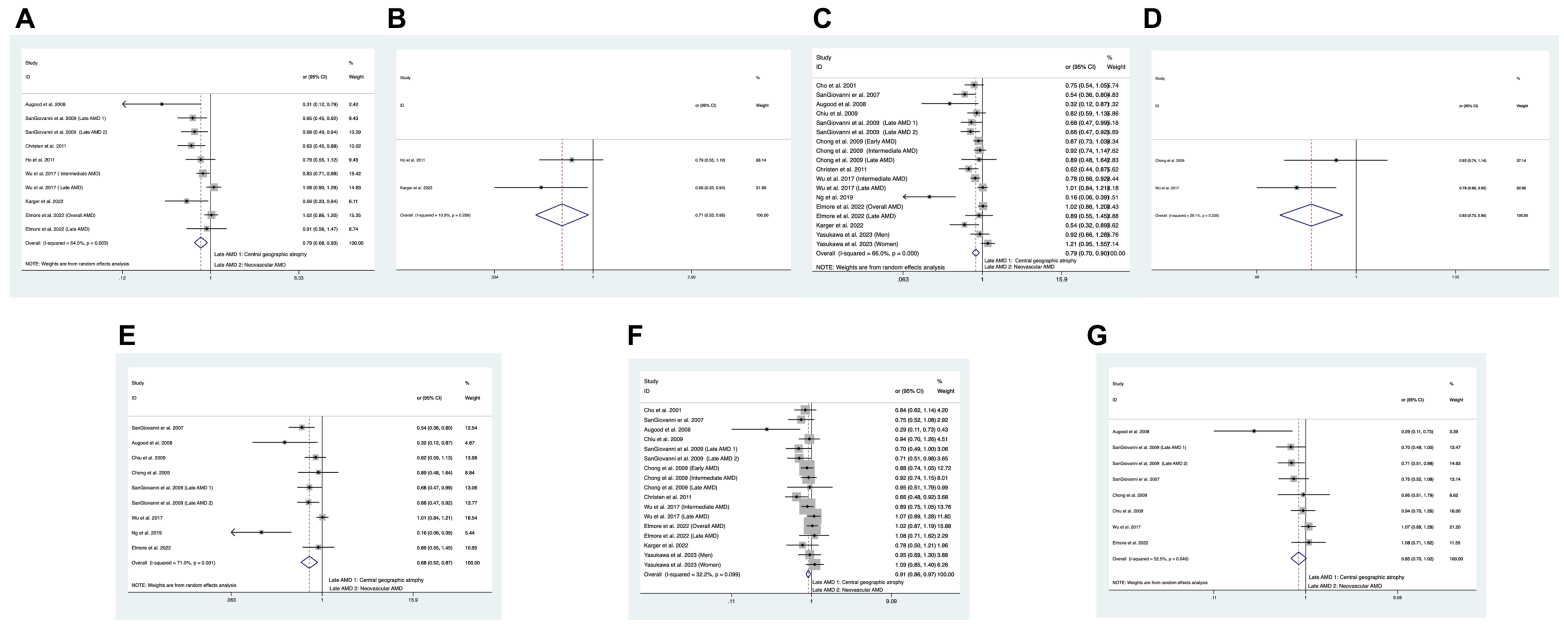
Forest plot of the odds risk (OR) of AMD for the highest vs. lowest level intake of DHA and EPA in studies. **(A)** The odds risk of combined intake of DHA and EPA and overall AMD; **(B)** the odds risk of combined intake of DHA and EPA and early AMD; **(C)** the odds risk of DHA and overall AMD; **(D)** the odds risk of DHA and intermediate AMD; **(E)** the odds risk of DHA and advanced AMD; **(F)** the odds risk of EPA and overall AMD; **(G)** the odds risk of EPA and advanced AMD.

In total, seven of the included studies analyzed the relationship between combined intake of DHA and EPA and the risk of overall AMD, and the statistical heterogeneity among the studies was large (I^2^ = 64.5%, P_H_ = 0.003) (11, 14, 17, 39, 40). We compared the high and low intake levels of DHA and EPA and observed a difference between the increased intake levels of DHA and EPA and the low-intake group in the risk of overall AMD (OR: 0.79; 95%CI: [0.67, 0.93]; *p* = 0.035) or early AMD (OR: 0.71; 95%CI: [0.53, 0.95]; *p* = 0.022) as shown in Figures 4A,B. In contrast, no difference was found between the two groups in the risk of advanced AMD.

In addition, 12 studies analyzed the relationship between DHA intake and the risk of overall AMD, and large statistical heterogeneity was observed among studies (I^2^ = 66.0%, P_H_ < 0.001) (11–21, 40). We compared the high and low intake levels of DHA and observed a difference in the risk of overall AMD (OR:0.80; 95%CI:[0.70, 0.90]; *p* < 0.001), intermediate AMD (OR:0.83; 95%CI:[0.73, 0.95]; *p* = 0.005), or advanced AMD (OR:0.68; 95%CI:[0.52, 0.87]; *p* = 0.003) between the increased intake of DHA and the low-intake group as shown in Figures 4C–E. In contrast, the increased intake of DHA was not related to the risk of early AMD.

Finally, 11 studies analyzed the relationship between EPA intake and the risk of overall AMD, and no statistical heterogeneity was observed among studies (I^2^ = 32.2%, P_H_ = 0.099) (11–15, 17–21, 40). We compared the high and low intake levels of EPA and a significant difference was found between the increased intake of EPA and the low-intake group in the risk of overall AMD (OR:0.91; 95%CI: [0.86, 0.97]; *p* = 0.004) and advanced AMD (OR:0.85; 95%CI: [0.70, 1.02]; *p* = 0.034) as shown in Figures 4F,G. In contrast, the increased intake of DHA was not related to the risk of early AMD or intermediate AMD.

#### 3.4.9 Oleic acid, linolenic acid, docosapentaenoic acid (DPA, C22:5n3)

In addition, two of the included studies analyzed the relationship between oleic acid intake and the risk of overall AMD, and the statistical heterogeneity among the studies was large (I^2^ = 88.6%, P_H_ = < 0.001) (16, 21). We compared the high and low intake levels of oleic acid and observed no difference in the risk of overall AMD and advanced AMD between the increased intake of oleic acid and the low-intake group.

Additionally, two studies analyzed the relationship between linolenic acid intake and the overall risk of AMD, with no statistical heterogeneity among the studies (I^2^ = 49.8%, P_H_ = 0.137) (18, 20). No difference was found in the risk of overall AMD between both groups.

Finally, two studies analyzed the relationship between DPA intake and the risk of overall AMD, and large statistical heterogeneity was observed among the studies (I^2^ = 93.8%, P_H_ = < 0.001) (13, 16). The high DPA intake did not influence the risk of overall AMD in comparison with the low-intake group.

#### 3.4.10 Omega-6 PUFAs family

In total, five of the included studies analyzed the association of intake of omega-6 PUFAs with the risk of overall AMD, and the statistical heterogeneity among the studies was large (I^2^ = 69.7%, P_H_ = 0.006) (13, 18, 22–24). We compared the high and low intake levels of omega-6 PUFAs and found that increasing the intake of omega-6 PUFAs did not affect the risk of overall AMD, early AMD, or intermediate AMD.

For other subgroups of the omega-6 family, six studies examined the relationship between linoleic acid intake and the risk of overall AMD, and large statistical heterogeneity was observed among studies (I^2^ = 0.0%, P_H_ = 0.911) (13, 15, 17, 20, 21, 32). No significant difference was found in reducing the overall AMD, early AMD, or advanced AMD between the high and low intake levels of linoleic acid.

In addition, seven studies examined the relationship between arachidonic acid intake and the risk of overall AMD, and large statistical heterogeneity was observed among studies (I^2^ = 72.7%, P_H_ = <0.001) (13, 15–17, 20, 21, 32). The high intake of arachidonic acid had no association with reduced overall AMD, early AMD, or advanced AMD compared to the low-intake group.

### 3.5 Sensitivity analysis and publication bias assessment results

**Figure 5:**
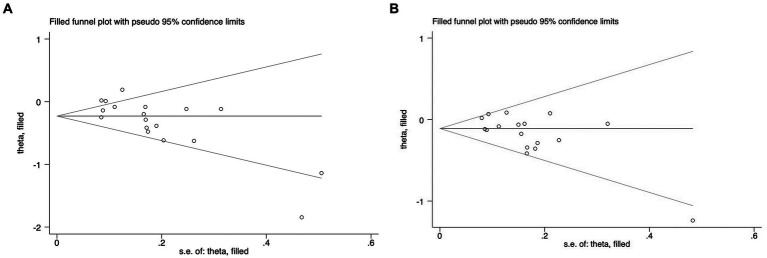
Trim-and-fill method for further verification of publication bias among studies.

For the studies on overall AMD in the above groups of fatty acids, sensitivity analysis was conducted by removing one study at a time. No individual study was found to affect the pooled effect size, indicating that the results were robust. We used Egger’s test to assess the publication bias for total fat, SFAs, MUFAs, PUFAs, omega-3 family, DHA, and EPA (Table 5). Egger’s test results suggested the presence of publication bias in DHA (*p* = 0.003) and EPA (*p* = 0.017). Therefore, we corrected the results using the trim-and-fill method and found that no new studies were added to the analysis, indicating that the existing publication bias did not affect the results of the study as shown in Figure 5. Other FA groups did not have publication bias.

**Table 5:** Results of the meta-analysis.

Fatty acid type			Number of studies	Sample size	Heterogeneity, I^2^, *p*	Model	OR, 95%CI	*P*	Egger, *P*
Total fat			10	8,248	59.1%, 0.001	Random	0.99(0.84,1.17)	0.881	0.71
		EA	5	2,332	45.2%, 0.104	Fixed	0.99(0.86,1.12)	0.82	N/A
		IM	2	2,697	0.0%, 0.828	Fixed	1.04(0.86,1.26)	0.707	N/A
		LA	5	279	0.0%, 0.822	Fixed	0.85(0.57,1.27)	0.426	N/A
Trans-fatty acid			6	3,515	63.6%, 0.005	Random	1.06(0.85,1.33)	0.061	N/A
		EA	4	2,389	62.1%, 0.048	Random	0.89(0.55,1.44)	0.628	N/A
		IM	2	1,111	65.9%, 0.087	Random	1.31(0,60,2.85)	0.5	N/A
		LA	3	358	0.0%, 0.811	Fixed	2.05(1.29,3.25)	0.002	N/A
Saturated fatty acid			14	8.936	55.5%, 0.001	Random	1.04(0.89,1.22)	0.591	0.343
		EA	8	2,533	72.5%, 0.001	Random	1.01(0.76,1.33)	0.795	N/A
		IM	3	4,898	63.9%, 0.063	Random	0.89(0.52,1.53)	0.675	N/A
		LA	7	1,065	29.6%, 0.202	Fixed	1.17(0.90,1.52)	0.238	N/A
Monounsaturated fatty acid			12	9,048	61.3%, < 0.001	Random	0.93(0.78,1.11)	0.422	0.513
		EA	6	4,485	68.2%, 0.008	Random	0.81(0.59,1.11)	0.195	N/A
		IM	2	2,697	91.6%, 0.001	Random	0.45(0.08,2.57)	0.366	N/A
		LA	6	1,035	56.3%, 0.043	Random	1.05(0.68,1.63)	0.827	N/A
Polyunsaturated fatty acid			11	6,715	53.4%, 0.006	Random	0.94(0.80,1.11)	0.452	0.702
		EA	7	3,175	55.8%, 0.035	Random	1.00(0.78,1.29)	0.98	N/A
		LA	5	445	72.6%, 0.006	Random	0.73(0.34,1.58)	0.429	N/A
Omega-3			10	7,912	68.3%, <0.001	Random	0.87(0.72,1.05)	0.148	0.612
		EA	5	1,926	0.0%, 0.461	Fixed	0.82(0.71,0.95)	0.008	N/A
		IM	3	2,898	82.1%, 0.004	Random	1.30(0.72,2.32)	0.383	N/A
		LA	6	497	76.4%, 0.001	Random	0.63(0.29,1.36)	0.241	N/A
	Long-chain omega-3		2	936	0.00%, 0.683	Fixed	0.67(0.51,0.88)	**0.004**	N/A
		LA	2	716	0.0%, 0.823	Fixed	0.60(0.42,0.87)	0.006	N/A
	DHA + EPA		7	5,341	64.5%, 0.003	Random	0.79(0.67,0.93)	**0.035**	N/A
		EA	2	731	10.9%, 0.289	Fixed	0.71(0.53,0.95)	**0.022**	N/A
		LA	7	2,985	66.2%, 0.007	Random	0.85(0.69,1.05)	0.135	N/A
	DHA		12	14,702	66%, 0.000	Random	0.80(0.70,0.90)	**0**	**0.003**
		EA	2	1,225	67.7%, 0.078	Random	0.76(0.46,1.15)	0.173	N/A
		IM	2	2,499	29.1%, 0.235	Fixed	0.83(0.73,0.95)	0.005	N/A
		LA	9	4,366	71.0%, 0.001	Random	0.68(0.52,0.87)	0.003	N/A
	EPA		11	15,603	32.2%, 0.099	Fixed	0.91(0.86,0.97)	**0.004**	**0.017**
		EA	2	2,135	0.00%, 0610	Fixed	0.87(0.73,1.02)	0.082	N/A
		IM	2	2,499	0.0%, 0.815	Fixed	0.90(0.79,1.03)	0.126	N/A
		LA	8	4,267	52.5%, 0.04	Random	0.85(0.70,1.02)	**0.034**	N/A
	Oleic acid		2	2,097	88.6%, 0.000	Random	1.51(0.91,2.51)	0.115	N/A
		LA	2	176	94.0%, 0.000	Random	3.82(0.31,46.92)	0.295	N/A
	Linolenic acid		2	2,923	49.8%, 0.137	Fixed	1.08(0.90,1.28)	0.407	N/A
	ALA		8	6,775	55%, 0.01	Random	1.01(0.89,1.14)	0.937	N/A
		EA	3	1,389	77.9%, 0.011	Random	1.07(0.67,1.71)	0.781	N/A
		IM	2	2,119	75.8%, 0.042	Random	1.10(0.81,1.49)	0.53	N/A
		LA	6	3,556	50.3%, 0.073	Random	0.93(0.74,1.18)	0.548	N/A
	DPA(C22:5n3)		2	334	93.8%, 0.000	Random	0.33(0.06,1.97)	0.225	N/A
Omega-6			5	5,211	69.7%, 0.006	Random	0.93(0.69,1.27)	0.661	N/A
		EA	2	2,193	0.00%, 0.594	Fixed	0.97(0.68,1.38)	0.865	N/A
		IM	2	1,988	92.9%, 0.000	Random	0.69(0.08,6.04)	0.739	N/A
	Linoleic acid		6	4,114	0.00%, 0.911	Fixed	0.95(0.87,1.04)	0.26	N/A
		EA	2	2,141	0.00%, 0.855	Fixed	0.91(0.76,1.08)	0.265	N/A
		LA	4	2,249	0.00%, 0.719	Fixed	1.01(0.79,1.29)	0.926	N/A
	Arachidonic acid		7	4,213	72.7%, 0.000	Random	1.09(0.90,1.33)	0.371	N/A
		EA	2	2,141	0.00%, 0.790	Fixed	0.86(0.74,1.00)	0.053	N/A
		LA	5	2,546	83.9%, 0.000	Random	1.61(0.82,3.14)	0.164	N/A

The bold values indicate *p*-values ≤ 0.05.

## 4 Discussion

We included 26 studies with 241,151 participants to summarize the relationship between various FA subtypes and AMD in different stages, providing the latest epidemiological evidence. The evidence in this meta-analysis was of high quality and showed that the high intake of omega-3 LCPUFAs, DHA, and EPA as well as the simultaneous intake of DHA and EPA lowered the risk of overall AMD. In contrast, the high and low intake levels of total fat, SFAs, MUFAs, PUFAs, and omega-6 showed no statistical significance in reducing the risk of overall AMD.

The high intake of omega-3 and simultaneous intake of DHA and EPA were statistically significant in reducing early AMD compared to the low-intake group. In addition, a high intake of DHA had a significant protective effect against risk factors for intermediate AMD compared to the low-intake group. Additionally, a high intake of omega-3 LCPUFA, DHA, and EPA significantly reduced the risk of advanced AMD in comparison with the low-intake group. It is worth noting that a high intake of TFAs increased the risk of advanced AMD, and previous meta-analyses have not reported this finding. Studies over the past decades have indicated that a higher intake of TFAs is positively related to a higher incidence of AMD and progression to advanced AMD, and the current meta-analysis has consistent results (21, 22, 34).

Several mechanisms have been suggested to underlie the protective effect of omega-3 LCPUFAs against the occurrence of AMD. The structure and function of the retina are highly dependent on FAs, and lipids make up one-third of the dry weight of the retina (41). FA is among the main nutrients in the human body. As a crucial component of the cell membrane’s lipid bilayer, FA participates in the formation of cholesteryl ester (CE) and functions in promoting membrane synthesis, immune signal transduction, gene expression regulation, and other systemic processes (8). In addition, FA can also be mobilized by cells as precursors of lipid mediators involved in many physiological processes, such as inflammation and neuroprotection (42). However, studies have shown that excessive lipid accumulation can promote the accumulation of advanced glycation end products and the activation of the protein kinase C pathway, which results in excessive reactive oxygen species, leading to oxidative stress and cytotoxic effects (43, 44). Accumulation of oxidation derived from lipoprotein in these extracellular deposits and pro-inflammatory lipids may trigger inflammation and innate immune responses through AMD pathophysiology-related complement activation, and the accumulated oxidative damage may lead to changes in the anatomy and physiology of photoreceptors, RPE, drusen, and chorion, thereby causing AMD (4).

PUFAs, including one of the ligands responsible for the activation of PPAR-α, inhibit NF-κB to produce strong anti-inflammatory effects (18). PUFAs contain several FA families, and the two primary families are omega-3 and omega-6. The α-linolenic acid (C18: 3 n-3; ALA) is the precursor of omega-3, and linoleic acid is that of omega-6. The α-linolenic acid and linoleic acid are metabolized to LCPUFAs that contain more double chains and/or carbon atoms *in vivo* after several steps, such as arachidonic acid (AA, C20: 4 n-6), EPA, and DHA, and this is of great significance for maintaining the function and life span of rod cells (42). EPA and DHA are major metabolites of the omega-3 family, with an estimated 8% of ALA converted to EPA and 1% to DHA (42, 45). EPA, as the precursor of DHA, can reduce blood lipids, avoid the formation of atherosclerotic plaque, and inhibit angiogenesis (46). DHA is the main component of membrane phospholipids, and the content of DHA is the highest in synapses and photoreceptors. DHA can regulate gene expression and fight against oxidative stress, inflammation, or apoptosis in retinal cells. DHA is therefore thought to be crucial for regulating inflammation (9, 15, 45, 47). The derivatives of EPA and DHA, such as lysins and neuroprotectins, can also protect photoreceptors from oxidative stress to fight against apoptosis and promote cell differentiation. Therefore, they all have anti-inflammatory properties (48).

The results of our research indicated that a high intake of LCPUFAs effectively lowered the risk of both overall AMD and progression to advanced AMD, and this was consistent with those of the Age-Related Eye Disease Study 2 (AREDS-2) and a 10-year cohort study (15, 32), indicating that a high intake of DHA and EPA may be able to lower the risk of overall AMD or progression to advanced AMD. In addition, a high dietary intake of omega-3 PUFA significantly reduced the risk of early AMD compared to the low-intake group but did not lower the risk of intermediate or advanced AMD as previously reported in some meta-analyses (7, 8). Some studies have hypothesized that this may be because the participants changed their overall diet after they had been diagnosed with AMD, leading to the fact that a high omega-3 PUFA intake did not significantly lower the risk of intermediate and advanced AMD (8).

According to the present meta-analysis, a high intake of DPA and EPA, respectively, reduced the risk of advanced AMD, and this result was consistent with the findings of Meng (49)and Jiang (7), but contrary to the findings of Zhong (8). This may be due to the inconsistency in the type of the studies included in their respective studies. Zhong included only prospective cohort studies and did not include cross-sectional and case–control studies, resulting in inconsistent results (8). Unlike the previously reported studies, this meta-analysis also analyzed the association of increased DHA intake with the risk of intermediate AMD, and a significant difference was found in the high DHA intake group in reducing the risk of intermediate AMD compared to the low-intake group. It is worth noting that simultaneous supplementation of DHA and EPA can effectively reduce the risk of overall and early AMD but cannot reduce the risk of advanced AMD. Among all the included studies, only Wu reported that the risk of advanced AMD had not been lowered for the simultaneous intake of EPA and DHA, we reviewed the study by Wu and found that simultaneous supplementation of DHA and EPA could lower the risk of intermediate AMD but could not lower the risk of advanced AMD. The specific reason may be related to the diet and health awareness of patients. In addition, Wu believed that reducing the incidence of intermediate AMD could ultimately achieve the purpose of reducing the risk of advanced AMD, and at least no harm has been reported in terms of simultaneous supplementation of DHA and EPA (40).

Many studies have shown that the anti-inflammatory mechanism of omega-3 is also related to its ability to inhibit the pro-inflammatory and pro-angiogenic effects of omega-6, and this is due to their competition for the same enzymes in the cyclooxygenase and lipoxygenase pathways (45, 50). Omega-6 produces prostaglandin E2, thromboxane A2, leukotriene B4, and other inflammatory substances after a series of metabolic processes *in vivo*, which further aggravates the oxidative stress of retinal cells and increases the development of AMD (42). This may explain the conflicting conclusions in the included studies that a high omega-6 intake has an increasing or decreasing effect on the risk of AMD. Among the included studies, Roh et al. found that omega-6 reduced the risk of AMD. They believed that this result was because their studies included not only Americans but also Portuguese, who mainly follow the Mediterranean diet (MD). In MD, high consumption of nuts is recommended, and nuts are rich in omega-6, which may slow the progression of AMD (22). However, omega-3 is also one type of rich nutrient in MD, and nuts are also reported to have rich EPA and DHA. Therefore, the MD increases the intake of omega-6 and omega-3 in the diet. This may have contributed to the protective effect of omega-6 on AMD in some studies (51, 52). In addition, Yasukawa also mentioned that the use of dietary supplements in American men and women was 74 and 79%, respectively, much higher than that in Japanese men and women, which was 30.2 and 38.2%, respectively. This may also lead to inconsistent results (18).

In clinical practice, ophthalmologists should provide different clinical recommendations based on the clinical classification of patients. For early-stage AMD patients, maintaining a healthy lifestyle is recommended, such as smoking cessation, a balanced diet (a diet rich in vegetables, fruits, fatty fish, and various foods rich in omega-3), and moderate physical activity. For patients with mid-stage AMD, in addition to a healthy lifestyle, supplementing antioxidant vitamins and minerals is recommended. According to the supplement formula proposed by AREDS-2, patients with mid-stage AMD should supplement with 500 mg of vitamin C, 400 IU of vitamin E, 10 mg of lutein, 2 mg of zeaxanthin, 80 mg of zinc oxide, and 2 mg of cupric oxide and regularly perform ophthalmologic examinations. For advanced patients, drug treatment is required in addition to the above-mentioned measures. For patients who have ever or currently smoked, lutein and zeaxanthin can be used, instead of β-carotene, to reduce the incidence of lung cancer, as β-carotene, abundant in the formula of AREDS-2, may increase the risk of lung cancer (53).

## 5 Limitations

First, the sample sizes of studies about some FAs, such as oleic acid, linolenic acid, DPA, and omega-6, are small, and this may lead to a less robust meta-analysis of these FAs, thereby limiting the interpretation of the results. Second, as some of the included studies were published years ago, there are some differences in the staging and diagnosis of AMD in different studies, and some studies did not differentiate AMD in different stages. Therefore, studies on the association of FAs with the risk of AMD in different stages are small in number, and this may lead to underestimation or overestimation of the relationship between intake of FAs and the risk of AMD. Third, the included studies were published within a long time span and involved different clinical factors, including age, country, and population, and this may lead to some biases. Fourth, in all the included studies, the FA intake was assessed using questionnaires that have many limitations, such as incorrect reporting of diet, and measurement errors are inevitable, and these factors may lead to errors in the relationship between FAs and the risk of AMD. Finally, the large number of cross-sectional and case–control studies (13 studies in total) included in this meta-analysis may have led to biases in data.

## 6 Conclusion

This meta-analysis provides evidence of high quality and showed that a high intake of LCPUFAs, DHA, EPA, or the simultaneous intake of DHA and EPA is strongly related to a decreased risk of overall AMD compared to the low-intake group. The simultaneous intake of high levels of DHA and EPA or high intake levels of omega-3 PUFA effectively decreases the risk of early AMD. In addition, a high intake of DHA has a strong association with a reduced risk of intermediate AMD. It is worth noting that a high intake of TFAs is significantly and positively correlated with advanced AMD, and the intake of TFAs should be reduced in daily diet. In future large-scale prospective studies, cross-sectional studies, or RCTs, more attention should be paid to the association of various intake levels of different FAs with the development and progression of intermediate AMD, to avoid the occurrence of advanced AMD as far as possible. Attention should also be paid to the association between AMD and the study population, dietary habits, and health awareness of the participants, and adjustments should be made accordingly, as this will greatly affect the study results.

## Data availability statement

The original contributions presented in the study are included in the article/Supplementary material, further inquiries can be directed to the corresponding author.

## Author contributions

YL: Writing – review & editing, Writing – original draft, Visualization, Validation, Methodology, Conceptualization. LL: Writing – review & editing, Investigation, Funding acquisition, Formal analysis, Data curation. LZ: Writing – review & editing, Software, Funding acquisition, Formal analysis, Data curation. QZ: Writing – review & editing, Writing – original draft, Supervision, Resources, Project administration, Conceptualization.
