# Supplementary material for: Association between fatty acid intake and age-related macular degeneration: a meta-analysis
Source: Front Nutr. 2024 Jun 26;11:1403987. doi: 10.3389/fnut.2024.1403987 (PMC11234253; doi:10.3389/fnut.2024.1403987)
Supplement: Supplementary file 1 [file Table_1.DOCX]

**Search strategies**

**Pubmed**

| Search number | Query | Results | |
| --- | --- | --- | --- |
| 5 | ((fish Mediterranean diet*[Title/Abstract]) OR ((Fatty Acid*[Title/Abstract]) OR (Aliphatic Acid*[Title/Abstract]))) AND (((Age Related Macular Degeneration*[Title/Abstract]) ) OR (Age Related Maculopath*[Title/Abstract])) | 363 | |
| 4 | (fish Mediterranean diet*[Title/Abstract]) OR ((Fatty Acid*[Title/Abstract]) OR (Aliphatic Acid*[Title/Abstract])) | 278,312 | |
| 3 | fish Mediterranean diet*[Title/Abstract] | 889 | |
| 2 | (Fatty Acid*[Title/Abstract]) OR (Aliphatic Acid*[Title/Abstract]) | 277,641 | |
| 1 | ((Age Related Macular Degeneration*[Title/Abstract]) ) OR (Age Related Maculopath*[Title/Abstract]) | 24,345 | |
|  |  |  |  |

**Cochrane**

| No. | Query | Results |
| --- | --- | --- |
| #1 | (Age Related Macular Degeneration*) OR (Age Related Maculopath*) | 3,842 |
| #2 | (Fatty Acid*) OR (Aliphatic Acid*) | 23,096 |
| #3 | fish Mediterranean diet* | 258 |
| #4 | #3 OR #2 | 23258 |
| #5 | #4 AND #1 | 115 |

**Embase**

| No. | Query | Results |
| --- | --- | --- |
| #66 | #1 AND #4 | 481 |
| #4 | #2 OR #3 | 322,254 |
| #3 | 'fish mediterranean diet*':ab,ti | 1 |
| #2 | 'fatty acid*':ab,ti OR 'aliphatic acid*':ab,ti | 322,254 |
| #1 | 'age related macular degeneration*':ab,ti OR 'age related maculopath*':ab,ti | 32,059 |

**Web of Science**

| # | Search Query | Results |
| --- | --- | --- |
| 8 | Age Related Maculopath* (Topic) OR Age Related Macular Degeneration* (Topic) and Preprint Citation Index (Exclude – Database) | 48,503 |
| 9 | Fatty Acid* (Topic) OR Aliphatic Acid* (Topic) and Preprint Citation Index (Exclude – Database) | 75,2873 |
| 10 | fish Mediterranean diet* (Topic) and Preprint Citation Index (Exclude – Database) | 6,974 |
| 11 | #9 OR #10 and Preprint Citation Index (Exclude – Database) | 75,8195 |
| 12 | #11 AND #8 and Preprint Citation Index (Exclude – Database) | 1,188 |
